# Applicability of electronic sphygmomanometer use in high-altitude areas according to the AAMI/ESH/ISO collaboration statement

**DOI:** 10.3389/fcvm.2023.1257444

**Published:** 2024-01-08

**Authors:** Xueting Liu, Runyu Ye, Xin Zhang, Wen Huang, Lirong Sun, Xingwei Huo, Xiaoping Chen

**Affiliations:** Department of Cardiology, West China Hospital of Sichuan University, Chengdu, China

**Keywords:** blood pressure determination, sphygmomanometers, oscillometry, altitude, validation studies as topic

## Abstract

**Objective:**

Mercury sphygmomanometer (MS) has now been less and less used and no new devices have been manufactured (according to Minamata convention 2013). The application of the electronic sphygmomanometer (ES) in clinical practice has become increasingly common. However, reliable evidence for the use of the ES in high-altitude areas remains scarce. The purpose of this study was to validate the applicability of the ES in high altitude areas.

**Methods:**

In Luhuo County, Sichuan Province, China, 3,400 m above the sea level, two trained physicians measured the blood pressure (BP) of participants using both the mercury sphygmomanometer and the ES. Pearson correlation analysis and paired T-test, respectively, were used to compare the correlation and the difference between the BP values measured by the two devices. The applicability of the ES in high-altitude areas was evaluated according to the validation standards of the 2018 Association for the Advancement of Medical Instrumentation/European Society of Hypertension/International Organization for Standardization (AAMI/ESH/ISO) Collaboration Statement.

**Results:**

In this study, 257 participants were included. There was a strong correlation between BP values measured by the two devices, with correlation coefficients for systolic blood pressure (SBP) and diastolic blood pressure (DBP) of 0.97 and 0.93, respectively. Compared with the MS, the ES tended to measure the subjects' DBP (76.21 ± 13.29 mmHg vs. 76.53 ± 14.07 mmHg; *P* = 0.557) accurately, but overestimate the SBP of the subjects (123.32 ± 22.25 mmHg vs. 121.34 ± 22.88 mmHg; *P *< 0.001) to some extent. The consistency of the two devices in the classification of normal BP, prehypertension, and hypertension was 88.9%, 80.7%, and 89.2%, respectively.

**Conclusions:**

In general, the utilization of ES at 3,400 m altitude successfully met the validation standards of the AAMI/ESH/ISO Collaboration Statement. The use of ES can be recommended at a high altitude, including up to 3,400 m. In addition, because the ES tended to overestimate SBP, we speculate that it may need to be calibrated in high-altitude areas.

## Introduction

Recent studies have reported that cardiovascular disease (CVD) is gradually surpassing cancer as the leading cause of death worldwide (1–3). Hypertension is associated with the strongest evidence for causation among all the risk factors for CVD (4), and it is also among the most important modifiable risk factors for all-cause morbidity and mortality (5). Hypertension is well known to have a high prevalence worldwide, including among the estimated 140 million people permanently living in high-altitude regions (>2,500 m) (6). A recent systematic review and meta-analysis revealed that the pooled prevalence of hypertension among the general population in high-altitude areas was as high as 33.0% (7). Therefore, it is particularly important to prevent and control hypertension in these high-altitude areas.

The accurate measurement of blood pressure (BP) is essential for the diagnosis and management of hypertension, and the use of validated and calibrated devices is critical for obtaining accurate BP measurements (8). These BP values are usually obtained by non-invasive BP measurement, which is an indirect estimation of BP. At present, the principal non-invasive methods of BP monitoring are manual auscultation with the mercury sphygmomanometer (MS) and via the oscillometry with an electronic sphygmomanometer (ES) (9). Manual auscultation records systolic blood pressure (SBP) and diastolic blood pressure (DBP) by the presence or absence of the Korotkoff sound (10). Unlike the manual auscultation method, the oscillometry measures only the mean arterial pressure; SBP and DBP are approximated from the measured data. As pressure in the cuff is increased (over SBP), the flow of blood is completely occluded. When released, blood flow through the brachial artery produces shock waves; the cuff pressure corresponding to the highest peak of these shock waves represents the mean arterial pressure. Based on the statistical results of numerous clinical tests, an algorithm has been established to find the SBP at the 0.45 peak and the DBP at the 0.75 peak (8, 11–13). There may be some slight differences in this algorithm between different manufacturers of ES, but the principle is the same. Notably, the effect of low atmospheric pressure environments at high altitudes on shock waves is unknown. Thus it remains unknown whether ES algorithms for low altitudes are applicable for high-altitude areas (14).

At present in China, there are two existing verification regulations for ES use, neither of which involves the use of ES in high-altitude areas. In the YY0670-2008 (Non-invasive Automatic Electronic Sphygmomanometer) standard, the applicable atmospheric pressure range of ES is 80 kPa–105 kPa, equivalent to the range between 300 meters below sea level and 1,900 m above sea level (15). In JJG692-2010 (Verification Regulations for Non-invasive Automatic Measurement by Sphygmomanometer), the default assumption is that the verification of the ES is performed at normal atmospheric pressure (16). Currently, manual auscultation is considered the gold standard of non-invasive BP measurement and is used in international validation standards (17, 18). However, the MS has been gradually withdrawn from clinical practice and is being replaced by the ES because of the former's shortcomings such as high requirements for observers, observer bias, and especially mercury toxicity and the serious effects of mercury pollution on the environment (19). With the development of the oscillometric method, the ES has become the most common non-invasive BP measurement device in recent years because of its convenience (13). The ES is also recommended by international guidelines for avoiding observer bias. Therefore, the ES plays an important role in hypertension management in high-altitude areas.

However, the applicability of the ES in high-altitude areas is still lacking in evidence (20). To date, only two studies have evaluated the consistency of the MS and the ES in high-altitude areas. In the study by Li et al., the SBP measured by the ES was significantly higher than the SBP reported by the MS, whereas the DBP measured by the ES and the MS showed no significant difference (21). Cho et al. reported no statistical difference in DBP and SBP measured by the MS and the ES (22). In the verification of the ES, Li et al. adopted a non-standardized, internally devised protocol and Cho et al. adopted the European Society of Hypertension International Protocol 2010 that no longer in use. These two studies were carried out at different altitudes and adopted different sphygmomanometer verification procedures, thus leading to inconsistent conclusions. The utilization of the ES in high-altitude areas remains controversial. Therefore, it is indispensable to further evaluate whether the ES is suitable for high-altitude areas.

## Methods

### Device

The ES we used is the A&D ES (UA-651A; Japan). According to the instruction manual, the applicable pressure of the ES is 70–105 kPa, equivalent to 300 m below sea level to 2,700 m above sea level. The sphygmomanometer comes with a standard-sized cuff applicable to upper arm circumferences ranging from 22 cm to 32 cm. According to verification regulation JJG692-2010 (16), we used a non-invasive BP analyzer (FLUKE PSim8; America) at a room temperature of 20°C, humidity of 78%, and atmospheric pressure of 99.5 kPa in the Equipment Department of West China Hospital of Sichuan University to test the consistency of the ES. At the same time, the MS was also sent to the Equipment Department for verification.

### Recruitment

This study was conducted in three towns in Luhuo County in Sichuan Province, China, at an average altitude of 3,400 m. We recruited participants who were older than 18 years old; all participants were required to have lived in the towns for at least 3 months. The exclusion criteria were: (1) age > 80 years old; (2) atrial fibrillation; (3) upper arm circumference < 22 cm or > 32 cm; and (4) pregnancy including preeclampsia (18). The AAMI/ESH/ISO Collaboration Statement recommend that verification of a BP monitor requires at least 85 participants (18). We ultimately enrolled 257 participants in this study. This research was approved by the Ethics Committee of West China Hospital, Sichuan University (Chengdu, China). During the recruitment, two physicians explained the study plan to the participants. The researchers stated to the participants that the information and data collected during the study were for research analysis only and would not be disclosed without permission. Participants willing to participate in the study will sign the authorization. All participants provided informed consent.

### Data collection

Two trained physicians collected the demographic information, including gender and age, via questionnaires, measured the height, weight, and upper arm circumference of the participants using the unified calibrated instruments, and recorded the basic heart rhythm of the subjects through the 12-lead electrocardiogram.

Before measurement, the participants were required to be in a sitting position at rest for at least 5 min. All participants underwent three consecutive BP measurements, with each measurement taken after a 1-minute period of relaxation. Participant BP was measured by the same arm simultaneous method. We connected the MS and the ES to the same cuff using a *Y*-shaped tube so that both the two devices had the same pressure with the cuff. The inflation and deflation of the cuff were controlled by the ES. Two trained physicians used a dual-head teaching stethoscope to simultaneously take readings of the MS and then recorded the results by themselves. During the measurement, the screen of the ES remained covered. After 3 measurements were completed, the ES measurement results were taken using its memory mode and transcribed. The three BP values measured by the MS were recorded as Reference BP (R)1, R2, and R3 in sequence. The three BP values measured by the ES were recorded as Test BP (T)1, T2, and T3 in sequence.

During the measurement, if the difference between the two physicians on the SBP and DBP of the MS was greater than 4 mmHg, an additional set of supplementary measurement was added, up to 4 additional measurements. During the measurement, if there was a difference in SBP > 12 mmHg or a difference in DBP > 8 mmHg in any reference BP measurement, the participant was excluded. If the Korotkoff sound was not clear during the MS measurements, the participant was excluded (17).

### Definitions

The reference BP was defined as the average of the BP readings of the two physicians. The test BP was defined as the BP readings of the ES. According to the 2018 ESC/ESH Guidelines for the Management of Arterial Hypertension (23), BP levels were classified as normal BP, prehypertension, and hypertension. Hypertension was defined as SBP ≥ 140 mmHg and/or DBP ≥ 90 mmHg.120 mmHg ≤ SBP < 140 mmHg and/or 80 mmHg ≤ DBP < 90 mmHg was considered prehypertension. Normal BP was defined as SBP < 120 mmHg and DBP < 80 mmHg.

### Statistical analysis

IBM SPSS Statistics 26 was used for statistical analysis. The continuous variables were described by mean ± SD, and the categorical variables were described by numbers and percentages (*n*, %). Pearson correlation analysis and paired *T*-test, respectively, were used to compare the correlation and the difference between the BP values measured by the two devices. *P *< 0.05 was considered statistically significant. For the absolute value of BP difference ≤ 10 mmHg, frequency and percentage were described. The standardized Bland–Altman scatter plot and figures were drawn using GraphPad Prism 8.0 software. The validation of the ES in high altitude areas was according to the AAMI/ESH/ISO Collaboration Statement: A device is considered acceptable if its estimated probability of a tolerable error (≤10 mmHg) is at least 85% and both the SBP and DBP difference (test vs. reference), and its standard deviation should pass criteria 1 and 2 of ANSI/AAMI/ISO 81060–2 (17, 18).

## Results

### Participant characteristics

At an average altitude of 3,400 m, a total of 269 participants were initially investigated; 8 participants were excluded according to the inclusion and exclusion criteria, and 4 were excluded because of their reference BP differences in SBP > 12 mmHg or DBP > 8 mmHg. Finally, 257 participants were included. Overall, the proportion of men (58.8%) was higher than that of women (41.2%). According to the requirements of the 2018 AAMI/ESH/ISO Collaboration Statement (18), the distribution of 257 participants' upper arm circumference fulfilled the criterion that the proportion of the upper arm circumference in the lower eighth use range of the cuff was 12.1% (meeting the criterion of 10%), 23% (20% criterion) in the lower quarter, 56.5% (40% criterion) in the lower half, 43.5% (40% criterion) in the upper half, 22.1% (20% criterion) in the upper quarter, and 11.2% (10% criterion) in the upper eighth. The distribution of reference BP also fulfilled the criteria. SBP ≤ 100 mmHg accounted for 9.7% (meeting the criterion of 5%), ≥140 mmHg accounted for 21.4% (20% criterion), and ≥160 mmHg was 7.8% (5% criterion). DBP ≤ 60 mmHg accounted for 11.3% (meeting the criterion of 5%), ≥85 mmHg accounted for 23.3% (20% criterion), and ≥100 mmHg was 5.4% (5% criterion), as shown in Table 1.

**Table 1:** Characteristics of the study participants.

	Mean ± SD/ frequency	Range/percentage (%)	Cumulative percentage (%)
Gender (male/female)	151/106	–	
Age (year)	46.12 ± 17.39	18–80	
Height (cm)	164.73 ± 8.53	140–185	
Weight (kg)	68.81 ± 12.94	42.2–122.8	
BMI (kg/m^2^)	25.31 ± 4.08	16.63–38.13	
Upper arm circumference (cm)	26.90 ± 2.69	22–32	
Upper arm circumference (cm)			
22.00–23.25	31	12.1	12.1
23.25–24.50	28	10.9	23
24.50–27.00	86	33.5	56.5
27.00–29.50	55	21.4	77.9
29.50–30.75	28	10.9	88.8
30.75–32.00	29	11.2	100
SBP (mmHg)			
≤100	25	9.7	9.7
100 < SBP < 140	177	68.9	78.6
140 ≤ SBP < 160	35	13.6	92.2
≥160	20	7.8	100
DBP (mmHg)			
≤60	29	11.3	11.3
60 < DBP < 85	168	65.4	76.7
85 ≤ DBP < 100	46	17.9	94.6
≥100	14	5.4	100

BMI (body mass index) = weight (kg)/height^2^(m^2^); SBP, systolic blood pressure; DBP, diastolic blood pressure.

### Correlation and difference between the BP values measured by the MS and the ES

**Figure 1:**
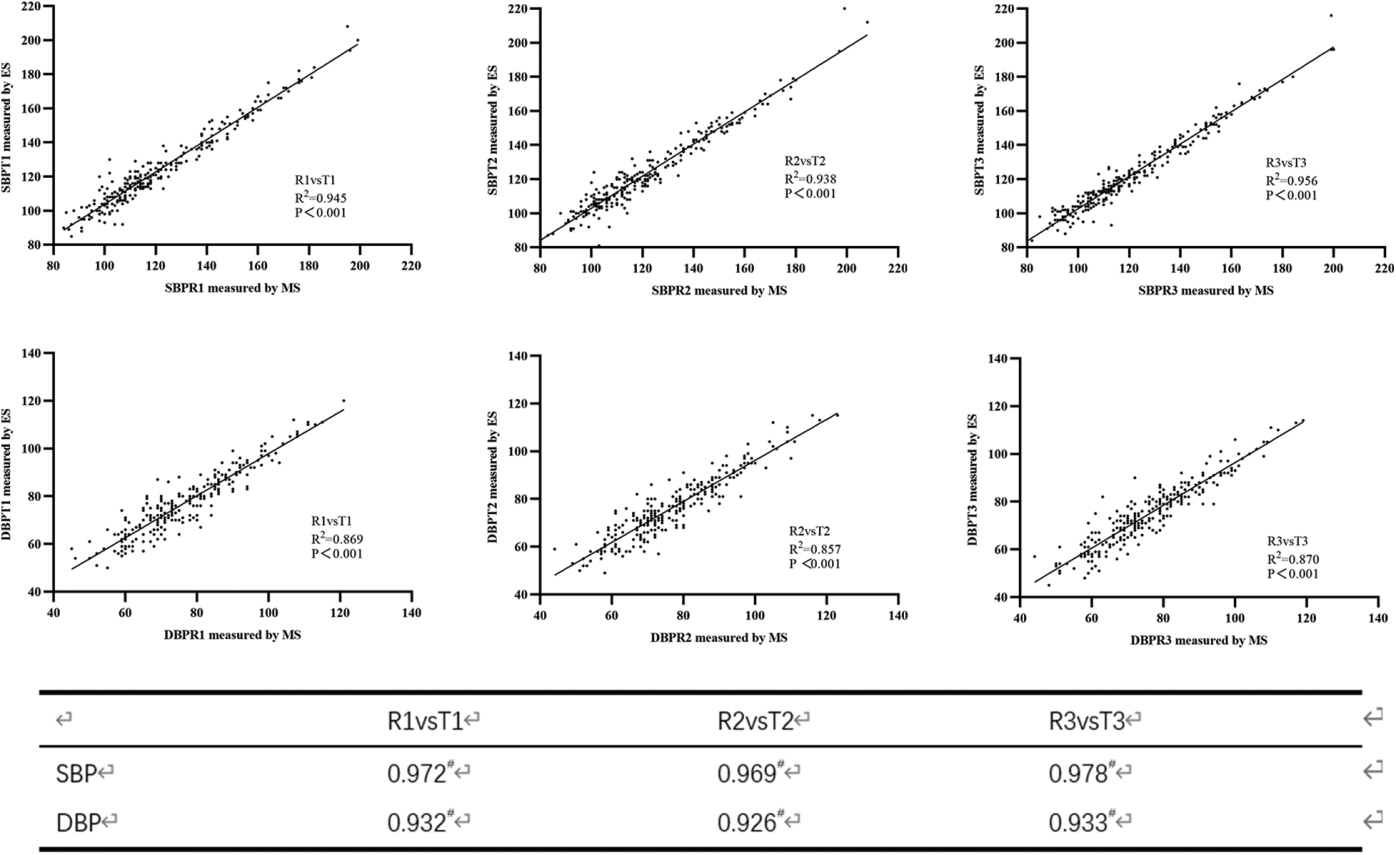
Scatter plots of blood pressure measured using the mercury sphygmomanometer (MS) and the electronic sphygmomanometer (ES) (upper three plots for systolic blood pressure (SBP) and lower three plots for diastolic blood pressure (DBP)).

Three groups of reference BPs and test BPs were obtained for every participant after three consecutive BP measurements. Figure 1 shows a strong linear relationship between BP values measured by the MS and the ES. The correlation coefficients for SBP measured by the ES and the MS were 0.972, 0.969, and 0.978, respectively; the correlation coefficients for DBP were 0.932, 0.926, and 0.933, respectively. In total, the mean SBP reported by the ES was higher than the SBP measured by the MS (123.32 ± 22.25 mmHg vs. 121.34 ± 22.88 mmHg; *P *< 0.001). The DBP reported by the ES showed little difference from the DBP measured by the MS (76.21 ± 13.29 mmHg vs. 76.53 ± 14.07 mmHg; *P *= 0.557). The mean differences between the BP values measured by the ES and MS were 1.98 mmHg [95% confidence interval (CI), 1.60–2.35 mmHg] for SBP and −0.32 mmHg (95% CI, −1.38 to 0.74 mmHg) for DBP (Table 2).

**Table 2:** Difference between reference and test BP.

	Reference BP (mmHg)	Test BP (mmHg)	Difference and 95%CI	*P* value
SBP				
R1vsT1	121.77 ± 23.13	124.41 ± 22.54	2.65 (1.98, 3.31)	<0.001*
R2vsT2	121.54 ± 22.90	123.24 ± 22.26	1.70 (1.00, 2.40)	<0.001*
R3vsT3	120.72 ± 22.67	122.30 ± 22.00	1.59 (1.00, 2.17)	<0.001*
Total	121.34 ± 22.88	123.32 ± 22.25	1.98 (1.60, 2.35)	<0.001*
DBP				
R1vsT1	76.44 ± 13.92	77.11 ± 13.14	0.67 (0.05, 1.29)	0.035
R2vsT2	76.77 ± 14.21	76.25 ± 13.17	−0.52 (−1.18, 0.14)	0.124
R3vsT3	76.37 ± 14.12	75.27 ± 13.54	−1.11 (−1.74, −0.48)	0.001*
Total	76.53 ± 14.07	76.21 ± 13.29	−0.32 (−1.38, 0.74)	0.557

Difference =** **test BP—reference BP; SBP, systolic blood pressure; DBP, diastolic blood pressure; R, reference blood pressure; *t*, test blood pressure.

* *P *< 0.01.

### Subgroup analysis

As shown in Table 3, we conducted further subgroup analysis to explore the difference between the BP values measured by the ES and the MS. According to the sex, the population were divided into male group (*n* = 151) and female group (*n* = 106). According to the age, the population were divided into three groups including 18 ≤ age < 40 group (*n* = 99), 40 ≤ age < 60 group (*n* = 94), and 60 ≤ age ≤ 80 group (*n* = 64). According to their BMI, the population were divided into standard group (*n* = 99), overweight group (*n* = 89) and obesity group (*n* = 69). The results of each subgroup were basically consistent with the overall results.

**Table 3:** Differences between reference and test BP in subgroups.

	Reference BP (mmHg)	Test BP (mmHg)	Difference and 95%CI	*P* value
SBP				
Male	122.80 ± 21.66	125.16 ± 21.01	2.37 (1.64, 3.09)	<0.001*
Female	119.27 ± 24.14	120.69 ± 23.30	1.43 (0.57, 2.28)	0.001*
18 ≤ age<40	108.78 ± 11.81	111.21 ± 11.41	2.43 (1.42, 3.44)	<0.001*
40 ≤ age<60	122.50 ± 21.13	124.06 ± 20.22	1.55 (0.85, 2.26)	<0.001*
60 ≤ age ≤ 80	139.07 ± 25.59	140.96 ± 24.96	1.89 (0.67, 3.11)	0.003*
Standard	114.26 ± 20.89	116.22 ± 20.02	1.95 (0.99, 2.93)	<0.001*
Overweight	120.26 ± 21.47	122.62 ± 21.18	2.36 (1.44, 3.28)	<0.001*
Obesity	132.81 ± 23.09	134.30 ± 22.19	1.49 (0.45, 2.53)	0.006*
DBP				
Male	78.62 ± 13.77	77.47 ± 12.97	−1.14 (−1.83, −0.46)	0.001*
Female	73.55 ± 13.71	74.41 ± 13.08	0.86 (−0.08, 1.81)	0.072
18 ≤ age<40	69.18 ± 11.03	68.64 ± 9.60	−0.54 (−1.54, 0.49)	0.292
40 ≤ age<60	78.71 ± 13.15	78.92 ± 12.51	0.21 (−0.80, 1.21)	0.686
60 ≤ age<80	84.68 ± 13.64	83.94 ± 12.60	−0.74 (−1.56, 0.08)	0.076
Standard	71.66 ± 13.82	71.46 ± 13.06	−0.21 (−1.10, 0.69)	0.65
Overweight	76.67 ± 12.81	76.44 ± 12.08	−0.23 (−1.27, 0.80)	0.656
Obesity	83.35 ± 13.06	82.61 ± 11.81	−0.73 (−1.87, 0.41)	0.203

Difference =** **BP measured by ES—BP measured by MS; SBP, systolic blood pressure; DBP, diastolic blood pressure; standard weight, BMI < 24; overweight, 24 ≤ BMI<28; obesity, BMI ≥ 28.

* *P* < 0.01.

### Consistency in classification of BP levels

As shown in Table 4, the percentages of consistency of the ES with the MS in the classification of normal BP, prehypertension and hypertension were 88.9%, 80.7% and 89.2%, respectively. The overall agreement rate between the MS and the ES was 87.2% (224/257).

**Table 4:** Classification consistency of the MS and the ES.

	Classification by MS measurement		
	Normal	Prehypertension	Hypertension
Classification by ES measurement			
Normal	120 (88.9%)	6 (10.5%)	0 (0%)
Prehypertension	15 (11.1%)	46 (80.7%)	7 (10.8%)
Hypertension	0 (0%)	5 (8.8%)	58 (89.2%)
Total	135 (100%)	57 (100%)	65(100%)

MS, mercury sphygmomanometer; ES, electronic sphygmomanometer.

### Validation procedure

**Figure 2:**
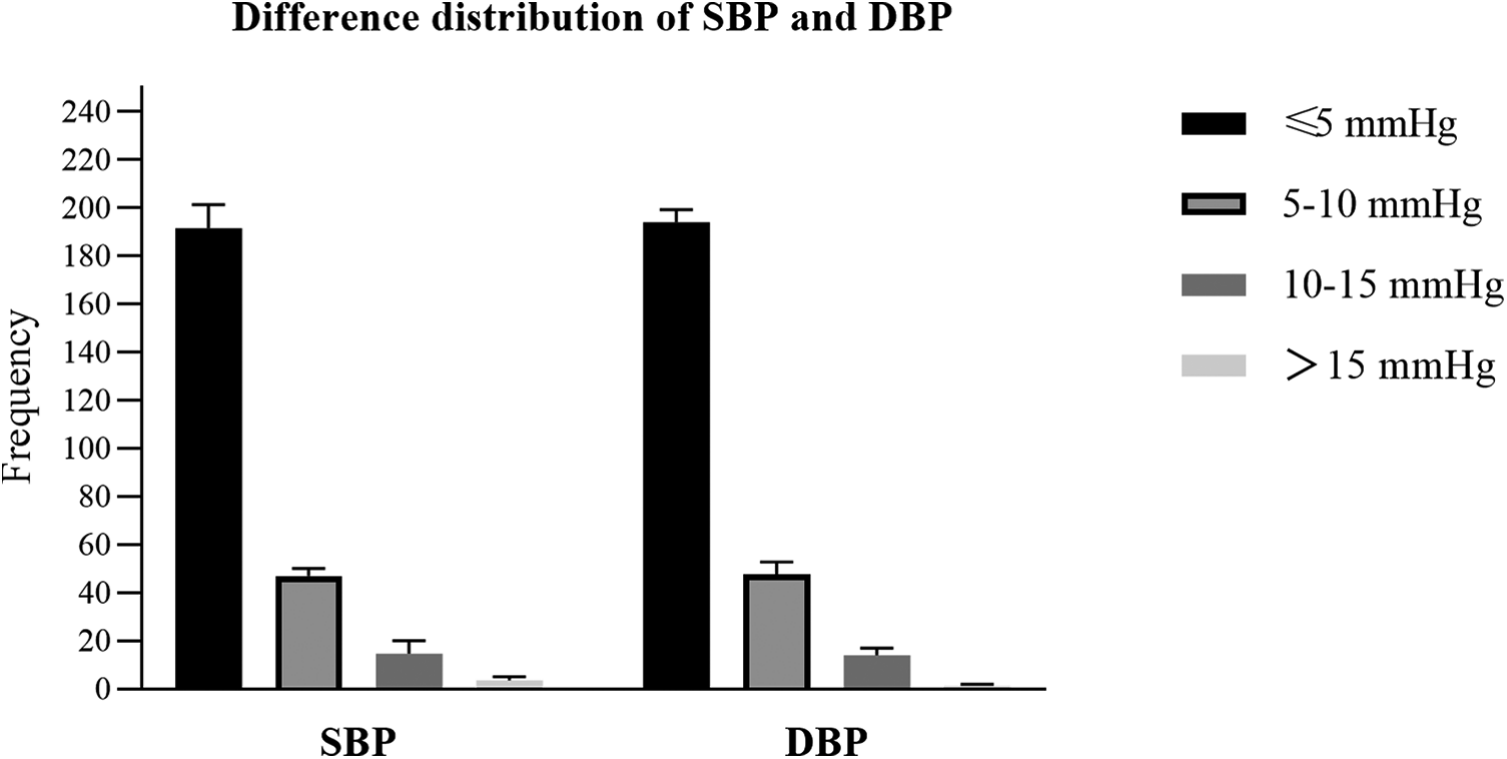
The differences between the test blood pressure (BP) measured by the electronic sphygmomanometer and the reference BP measured using the mercury sphygmomanometer were divided into four groups (≤5 mmHg, 5–10 mmHg, 10–15 mmHg, >15 mmHg). The y-axis represents the frenquecy of each group.

**Figure 3:**
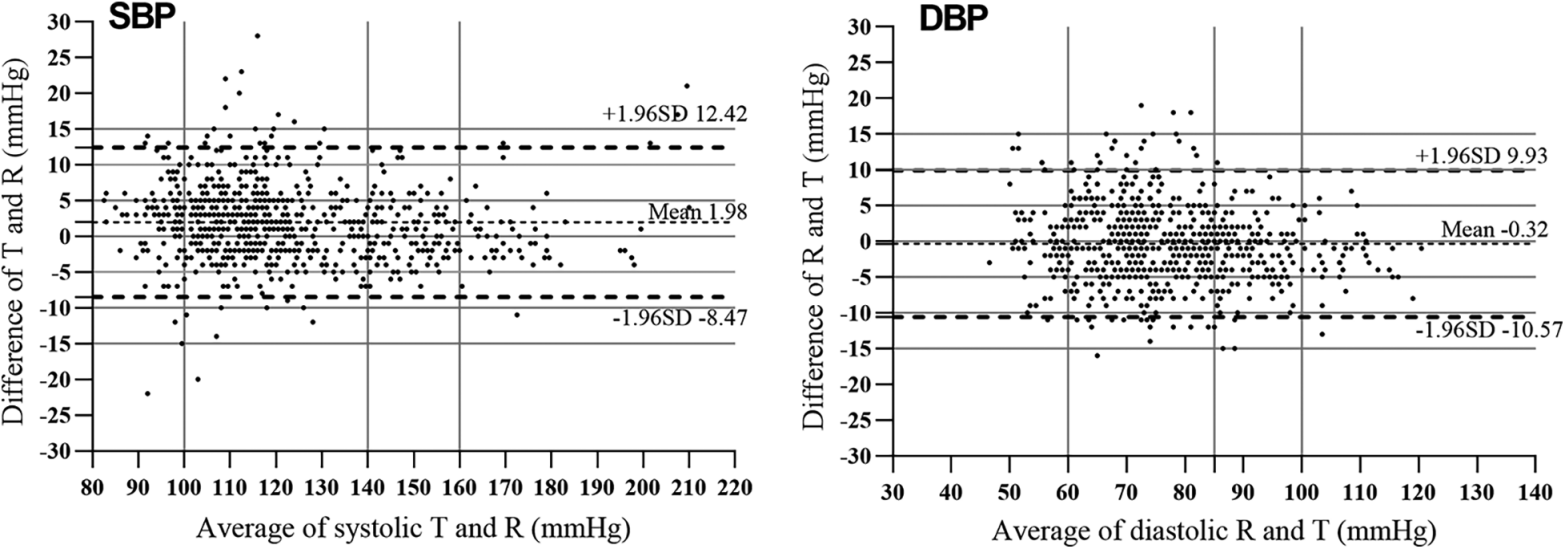
Bland–Altman plots for systolic blood pressure (SBP) and diastolic blood pressure (DBP) measured using the mercury sphygmomanometer (MS) and the electronic sphygmomanometer (ES). The average blood pressure values are shown against differences between MS and ES measurements. The solid horizontal line is the mean difference and the two dotted horizontal lines are ± 1.96 SD from the mean difference.

The differences between the test BP and the reference BP were divided into four groups (≤5 mmHg, 5–10 mmHg, 10–15 mmHg, >15 mmHg). Overall, SBP with a difference of <10 mmHg between the reference and the test accounted for 92.9% of the total, and DBP with a difference of <10 mmHg between the reference and the test accounted for 94.0% of the total (Figure 2). The mean and SD of differences between each pair of the reference BP and the test BP were 1.97 ± 5.33 mmHg for SBP and −0.32 ± 5.23 mmHg for DBP. The mean and SD of differences between the participants' average reference BP and test BP were 1.98 ± 4.49 mmHg for SBP and −0.32 ± 2.62 mmHg for DBP. As a result, the ES satisfied Criterion 1 and Criterion 2 in the ANSI/AAMI/ISO 81060-2:2018 (17) (Table 5). Lastly, we included 257 participants with a total of 771 sets of BP values in the standardized Bland–Altman scatterplots (Figure 3). A total of 37 points (4.8%) in the SBP and 47 points (6.1%) in the DBP were outside the limits of agreement (LoA) 95% CI. Such an error is acceptable in BP measurement.

**Table 5:** Validation results in accordance with criterion 1 and criterion 2 of the guidelines.

	Mean error of measurement	SD	Result
Criterion 1			
Requirement	≤5 mmHg	≤8 mmHg	
SBP	1.97	5.33	Pass
DBP	0.32	5.23	Pass
Criterion 2			
Requirement	≤5 mmHg	≤8 mmHg	
SBP	1.98	4.49	Pass
DBP	0.32	2.62	Pass

BMI(body mass index) = weight (kg)/height^2^(m^2^); SBP, systolic blood pressure; DBP, diastolic blood pressure.

## Discussion

Our study showed that in high altitude areas, the BP values measured by the MS and the ES are strongly correlated, with correlation coefficients for SBP and DBP of 0.97 and 0.93. Meanwhile, the ES tended to overestimate the SBP but measured DBP accurately. The mean differences between the BP values measured by the ES and the MS were 1.98 mmHg (95% CI, 1.60 to 2.35 mmHg) for SBP and −0.32 mmHg (95%CI, −1.38 to 0.74 mmHg) for DBP. Nevertheless, the mean difference between the reference SBP and the test SBP was just 1.98 mmHg, a difference with little clinical significance. The results of further subgroup analysis were basically consistent with the overall. In addition, the ES passed the detection level specified by the AAMI/ESH/ISO Collaboration Statement and therefore can be recommended for use at high altitude, including up to 3,400 m.

Only two previous studies have investigated the utilization of the ES in high altitude areas. The two studies' results showed a high degree of agreement for DBP when compared with MS at high altitudes. However, the degree of such agreement for SBP is not consistent (20). Cho's study was conducted in Lhasa, Tibet, with an average altitude of 3,650 m (22). This study reported no statistical difference between the differences of SBP (1.0 ± 5.9 mmHg) and DBP (3.1 ± 4.6 mmHg) measured by the ES and the MS (*P* value was not specified). This may be because of that study's small sample size of 33 subjects. Li's study was conducted in Dangxiong County, Tibet, China (4,300 m), and concluded that the ES could provide an accurate measurement of DBP (−0.4 ± 3.9 mmHg difference), but required a simple calibration to correct for overestimating SBP (5.8 ± 4.7 mmHg difference) (21). In our study, the mean differences between the BP values measured by the ES and the MS were 1.98 ± 0.28 mmHg for SBP and −0.32 ± 0.37 mmHg for DBP. Both our study and Li's study adopted the same-arm-simultaneously method of measuring BP. In terms of the difference between the BP values measured by the ES and the MS, our findings were similar to Li's study, though the errors in the SBP and the DBP in their study were slightly larger than ours. This may be because of the higher altitude of Dangxiong (4,300 m).

An ES identifies the signal of the shock waves created by the water hammer effect (24), then calculates the SBP and the DBP using an algorithm (11). In fluid mechanics, the shock waves are usually related to the density and speed of the fluid. However, human blood vessels are elastic, unlike ordinary engineering pipes. Whether the shock waves in the blood vessels are affected by atmospheric pressure remains open to discussion. According to our study results, we speculated that the algorithm of the ES in lower-altitude areas is not fully applicable to high-altitude areas. The ES verified in lower areas would produce certain errors in BP measurement when used in higher plateau areas; as the altitude increases, this error is likely to increase. However, in our study, the error at the high altitude of 3,400 m was too small to have clinical significance. Thus, the ES can be recommended for use at a high altitude up to 3,400 m.

In terms of validation procedures, Li's study included 85 participants using convenience sampling, but this sampling method limited the applicability of the findings to a wider population (21). In addition, Li's study verified the applicability of ES in high-altitude areas by an internally devised protocol instead of a standardized international verification procedure. Hence, the verification results of that study were not standardized and dependable. Cho's study included only 33 subjects, and therefore the evidence of the conclusion is insufficient (22). In fact, a study with a sample size of 35 is inadequate for evaluating a moderate accuracy device; at least 85 subjects are required (18).

The present study has several strengths. First, compared with previous studies, we further evaluated the applicability of the ES in high-altitude areas according to the latest AAMI/ESH/ISO Collaboration Statement. Second, we enrolled a total of 257 cases at an average altitude of 3,400 m, with strict inclusion and exclusion criteria. Further subgroup analysis also increases the reliability and applicability of our findings. In addition, our study also preliminarily discussed a possible mechanism of the limitations of ES use in high-altitude areas, which may provide evidence for the further improvement of oscillometric methods.

Despite these strengths, the limitations of our study are as follows. First, the participants' BP measurements were obtained using simultaneous arm measurement rather than the same-arm discontinuous measurement described in the AAMI/ESH/ISO Collaboration Statement. While simultaneous arm measurement ensures that the BP readings from both devices have the same pressure source, minimizing measurement error caused by BP variation (21), the drawback of simultaneous arm measurement is that the vent rate is controlled by the ES. The vent rate is always more than 2 mmHg/s, which may cause a reading error by the MS. In addition, the average altitude selected in this study was 3,400 m, but there are people living above 4,500 m or even higher. Hence the applicability of ES in very-high-altitude areas remains to be further verified.

## Conclusion

At an altitude of 3,400 m, the measurement results of the ES and the MS are strongly correlated. Meanwhile, compared with the BP values measured by the MS, the ES measured the DBP accurately but tended to overestimate the SBP. The ES showed good consistency with the MS in distinguishing normal BP, hypertension and prehypertension. The ES passed the validation criteria specified by the AAMI/ESH/ISO Collaboration Statement and therefore can be recommended for use at a high altitude up to 3,400 m.

## Data Availability

The original contributions presented in the study are included in the article/Supplementary Materials, further inquiries can be directed to the corresponding author.
